# Perception of simulation-based education among nursing and midwifery students in Tanzania: a qualitative study

**DOI:** 10.1186/s41077-025-00339-1

**Published:** 2025-03-08

**Authors:** Rosemary M. Malya, Michael J. Mahande, Kristin H. Urstad, Jane J. Rogathi, Bodil Bø

**Affiliations:** 1 Kilimanjaro Christian Medical University College, Moshi, Tanzania; 2 Kilimanjaro Christian Medical Centre, Moshi, Tanzania; 3 University of Stavanger, Stavanger, Norway

**Keywords:** Simulation-based education, Perception, Low-income context, Nursing and midwifery students, Tanzania

## Abstract

**Background:**

While many nursing programs in developed countries have implemented simulation-based education as a pedagogic method of teaching, implementation of simulation in developing countries like Tanzania is rare. Traditional methods of auditorium lectures are widely conducted in low-income nursing and midwifery education institutions. Such pedagogy provides students with theoretical knowledge yet with limited hands-on exposure for clinical skills, which might affect the professional integration of students and quality care delivery. This study explored perceptions of simulation-based education among diploma nursing students and midwifery students in one of the urban nursing schools in Tanzania.

**Method:**

An exploratory qualitative study design was employed. Thirty-four nursing and midwifery students who had experience with simulation-based education were selected purposively to participate in focus group interviews. Data was collected in July 2023. Data analysis was conducted based on Graneheim and Lundman’s content analysis approach.

**Results:**

Two major themes emerged from the analysis: (1) Strengthened confidence through practice in a safe teaching environment. This theme included three sub-themes: (i) Increased overall confidence, (ii) reduced fear through practice in a safe environment, and (iii) enhanced knowledge and skills in procedures and equipment. The second theme was as follows: (2) Enhanced critical thinking and reasoning in debriefing and included two sub-themes: (i) Integrating theory into practice, (ii) communication in neonatal emergency management.

**Conclusion:**

Nursing and midwifery students perceived simulation-based education as an effective method to prepare for clinical practice and quality neonatal care. Introducing simulation-based education in nursing education may benefit students’ learning and strengthen the sustainability of skilled healthcare providers in low-income contexts where resources are scarce. Further research is needed to assess whether students can transfer knowledge into clinical skills practice.

## Introduction

Simulation-based education (SBE) has been a vital component of nursing education worldwide for bridging the theory–practice gap through realistic simulated scenarios [1]. Previous studies indicate that SBE improves nursing students’ knowledge, confidence, skills, critical thinking, and reasoning in patient care [2–6].

While nursing programs in developed countries widely implement SBE as a method of teaching [7, 8], its implementation in low-income settings remains limited. Traditional methods of auditorium lectures are widely used in low-resource contexts, often providing minimal hands-on exposure of clinical skills [9]. In Tanzania, the nursing and midwifery curriculum was last reviewed in 2017, with simulation itemized as a teaching method. Few studies are available from Tanzania on SBE in nursing education institution settings. Evidence on the perception of simulation among nursing and midwifery students is minimal [10–13].

In the context of initial neonatal care (INC), various studies by Ersdal et al. [13], Mduma et al. [12], Matterson et al. [14], and Vadla et al. [15] have shown that SBE has improved health care practitioners’ (HCP) skills in neonatal care [12, 13].

In Tanzania, neonatal mortality rate was 24 deaths per 1000 live births in 2022 [16], exceeding the Sustainable Developmental Goal target of less than 12 deaths per 1000 live births by 2030 [13, 14]. Studies show that gaps persist in pre-service education [10, 16]. Inadequate INC competencies among HCPs may potentially lead to increased rates of neonatal deaths in low-income contexts [17–20]. According to the World Health Organization (WHO) [21] and a Lancet report [22], competent HCPs are essential for quality care including neonatal outcomes. SBE offers opportunities to build procedural competencies, enhance communication skills, and bridge the knowledge-practice gap, particularly in neonatal emergencies [23–25]. A previous study from a Tanzanian context reports how SBE improved nursing students’ confidence and competence in practice on a general level [10].

Jeffries [26] defines simulation as activities that mimic the reality of a clinical environment and are designed to demonstrate procedures, decision-making, and critical thinking through techniques such as role-playing and the use of devices such as interactive videos or mannequins. Simulation-based education (SBE) is performed in a controlled environment where facilitators engage students through three phases: [1] briefing, [2] scenario simulation, and [3] debriefing [27]. During debriefing, facilitators may follow Gibbs reflective learning cycle [28], to enhance learning [27, 29].

This structured approach facilitates critical reflection and prepares students for real-life clinical challenges [30, 31]. Despite the benefits of SBE shown in much of the literature, studies on nursing students in Tanzania are limited, with most focusing on emergency management among HCPs [10–13, 32, 33].

Therefore, this study aimed to explore the perception of SBE among diploma nursing and midwifery students in Tanzania on the topic of initial neonatal care.

## Methods

### Design and participants

The study used a qualitative descriptive design to explore the perception of SBE among diploma nursing and midwifery students in Tanzania. A total of 45 nursing students who had experience with SBE were invited for focus group discussions (FGD). Purposively, 34 nursing students were recruited to participate in the study [34].

### Simulation sessions

The SBE described in this study was conducted in June 2023, involving 45 s year diploma nursing and midwifery students. These students were participants in a quasi-experimental study focusing on simulation in the topic of “initial neonatal care.” According to the nursing and midwifery curriculum, the topic “initial neonatal care” should be taught through an auditorium lecture prior to clinical placements. In the previous study, this topic was covered in a 2-h lecture by the research investigator followed by SBE. During the SBE, low-fidelity simulators such as Baby Neonatalie were utilized for the scenarios. The students received two different initial neonatal care cases: [1] Scenario to a term normal healthy breathing neonate born by spontaneous vaginal delivery (SVD), and [2] scenario to a term neonate born by SVD who failed to initiate and sustain breathing spontaneously. The neonate had apnea and muscle weakness.

SBE sessions were conducted in line with Jeffries theory [26]. To create a relaxed and safe environment, the facilitator briefed the students about the purpose of the SBE and emphasized that the focus is on learning and not on assessment. The students were moreover briefed on objectives, expected outcomes, observer roles, and the subsequent debriefing session. Relevant resuscitative equipment for the scenario, such as Ambu-bag and mask, and how the equipment worked, were presented to assist students in achieving their learning objectives [35]. The first simulation scenario lasted 8 min, while the second lasted 5 min. The simulated scenarios were followed by debriefing sessions that were conducted by the facilitator based on Gibb’s (1988) reflective learning cycle [28]. The facilitator was trained as a facilitator of SBE. Each simulation session lasted approximately 50 min. All SBEs were conducted in the skills laboratory at the faculty of nursing. All students participated in both SBE cases with a focus on initial neonatal care.

### Data collection and instrument

The data was collected on 10th–14th July 2023 through focus group discussions (FGD) using an interview guide. A pilot study was conducted among four second year diploma nursing and midwifery students who had previous experience with SBE, to ensure that the interview guide was clear and understood by the students. The discussions were carried out 1 week after the SBE as a recall period within 2 weeks is considered to reduce risks of recall bias associated with the length of the recall period [36]. The interview guide was developed by the first researcher, consisted of questions regarding the students’ perception of SBE overall, their experience of the first time practicing SBE, different phases of simulation, and their opinions about SBE as a method of teaching in nursing and midwifery education. The focus group discussions were conducted in the nursing skills laboratory at the university campus. The skills laboratory has a comfortable atmosphere free from distractions enabling participants to openly share their experiences. The discussions were audio-recorded to capture interviews verbatim, accuracy in data collection and transcription. The discussions were conducted in English language based on students’ request because they found it easier to pronounce perceptions of the SBE experience in English rather than using Swahili language. Two researchers conducted the FGD. The first researcher facilitated the discussion, while the research assistant wrote notes. Both the researcher and the research assistant are university lecturers with experience in qualitative research methods. The participants, on the other hand, were students from a diploma nursing school who had no direct teaching or supervisory relationships with the researchers. This lack of a hierarchical relationship potentially minimized power dynamics, encouraging the participants to openly share their perceptions of SBE. Participants were interviewed until data saturation was reached, with no new relevant information emerging from additional participants [33].

### Trustworthiness

The study’s trustworthiness can be considered through the principles of credibility, confirmability, dependability, and transferability criteria [37]. Credibility involves selecting an appropriate study design, participants, data collection procedure, and data analysis process. Credibility was assured through a detailed description of the research setting, informants, researcher reflexivity, and data saturation [37]. Confirmability or external auditing of data was ensured by checking and rechecking data from collection to analysis and through discussion and consensus among the authors on the accuracy of themes and sub-themes [38]. Dependability ensures data stability across different stages. This was achieved through detailed description of informant’s characteristics and recruitment method [39]. Transferability was enhanced by providing details of the study site and informants, as well as a description of data collection, analysis, and results [40].

### Data analysis

Data analysis was conducted using Graneheim and Lundman’s content analysis [37]. The discussions were transcribed verbatim by the first investigator. Then two researchers independently read and reviewed the transcripts to become familiar with the data and commence initial analysis [39]. The two researchers cross-check interpretations and validate findings to increase the validity of the data. Regular team meetings were held to discuss discrepancies in coding or interpretation, refine coding frameworks, and resolve disagreements. The main segments of the data were designated “meaning units” which were condensed by shortening the texts while preserving their meaning. The condensed “meaning units” were interpreted and labeled with codes, which were grouped into different levels of categories to identify similarities and differences within and between categories to identify sub-themes and themes [37].

## Results

Thirty-four participants were recruited into the study. Participants were divided into three groups. Groups one and two included 11 informants each, and group three included 12 informants. Focus group discussions were 45–50 min in length.

Among 34 participants enrolled in the study, the majority 19 (55.6%) were female while 15 (44.1%) were male, with their age ranges from 19 to 25 years. Most participants 31 (91.2%) had form four secondary education at college entry, while 3 (8.8%) had form six secondary education. The content analysis results are presented below, organized into two major themes and their respective sub-themes (Table 1).

**Table 1:** Themes and sub-themes emerged from the analysis

**Theme**	Sub-themes
Theme 1: Strengthened confidence through practice in a safe teaching environment	(i) Increased overall confidence (ii) Reduced fear through practice (iii) Enhanced knowledge and skills in procedures and equipment
Theme 2: Enhanced critical thinking and reasoning in debriefing	(i) Integrating theory into practice (ii) Communication in neonatal emergency management

### Strengthened confidence through practice in safe teaching environment

The first main theme focuses on how students experienced SBE to strengthen their confidence through practice in a safe teaching environment. This overall theme is presented through the three sub-themes that emerged from the analysis (see Table 1).

#### I. Increased overall confidence

The students appreciated the training opportunity on clinical procedures and more complex cases outside the clinical arena, which was something they barely had done before. Students also appreciated that the facilitator briefly explained that the SBE was for learning and not for examining and assessment made the setting safe and more free of nervousness.

One informant expressed that the SBE “prepares us mentally and physically on how to practice and it gain us confidence” (FGD-2, R8). A vital perception that motivated the students for learning was that the SBE replicated recognizable clinical scenarios, as expressed by the following informant:

[….] During simulation in scenario, I gained more experience and confidence because the simulation reflects the reality of what we do in clinical areas. So, what we learned from simulation in the school has no any difference from the reality once we are in clinical areas (FGD-1, R5).

The students perceived that repeated simulation practices improved their confidence and performance in procedures that occurred quite rarely in clinical settings. As such, the students emphasized the importance of repeated training for becoming more competent and confident nurses. This repetitious training made students more effective in their performance as they knew what and how to carry it out.

One informant said:

Simulation has improved my performance and confidence in the clinical setting by increasing the speed of performing procedures. Previously, I lacked confidence in my practice, but attending the simulation sessions where we practice repeatedly, my confidence has really improved (FGD-3, R2).

#### II. Reduced fear through practice

The students emphasized the benefits of conducting SBE before attending patients as SBE provided a setting where mistakes could be made and corrected without harming patients, making students relaxed and safe in the teaching environment. The students expressed how they found SBE to prevent unnecessary injury to patients stated by this informant:

Simulation learning helps in maintaining safety of the baby because at the first time of simulation I found myself doing a lot of mistakes in the clinical scenarios. Now, in the clinical setting I am doing better because the mistakes have been corrected in the simulation process (FGD-2, R9).

The students specifically feared making mistakes in neonatal care, where mistakes could have serious consequences for neonates. They said that SBE reduced this fear. Simulation helped reduced fear of causing harm to neonates while providing care on them. One informant reflected upon how (s)he was worried to practice neonatal care before simulation, and said:

When I took part in simulation for the first time, I was worried because I didn't have enough experience of practicing well in initial neonatal care. After I did it in simulation many times, I gained confidence and so it built up my confidence to practice to a real neonate in the hospital setting (FGD-1, R6).

#### III. Enhanced knowledge and skills in procedures and equipment

The last sub-theme focuses on how the students expressed that SBE enhanced their knowledge in procedures and best practice with equipment. They felt confident and moreover became more aware of the equipment and neonatal care practices which again strengthened their practical skills in initial neonatal care. The students reported that they specifically were afraid of using resuscitation equipment to help babe breathe—for neonatal resuscitation while in the clinical settings—something they had little experience in. One student reflected upon how (s)he didn’t know how to practice with the APGAR score chart and Ambu-bag before simulation and said:

Before simulation, I didn’t know how to score the baby, nor how to use the resuscitation equipment like Ambu-bag […] After coming over here in simulation, I got the knowledge on how to use Ambu-bag, and I am not afraid of anything from the reality of newborn care (FGI-3, R-4).

Another informant said:

At first when I saw in the clinical area, I was afraid to use it, but when I came and practiced through simulation, I managed to use the Ambu-bag during neonatal resuscitation[.…]when I went back to the clinical area, I didn't think that it was only for the nurses professional who could use Ambu-bag during resuscitation, but I could also use it and manage to perform resuscitation ona newborn (FGI −2, R-6).

The reduction of fear of causing injury to neonates also involved the students’ confidence on an overall agenda. One informant said this:

Simulation needs to be included in the nursing and midwifery curriculum as it creates confidence and perfection in performing many other procedures. And for now, we are performing only in the initial neonatal care, but if it will be included in the curriculum, it will help in doing correctly many more procedures than that in the initial neonatal care (FGD −2, R4).

### Enhanced critical thinking and reasoning during debriefing

The second main theme includes how the students expressed that SBE enhanced their critical thinking and reasoning during debriefing session. This theme included two sub-themes (see Table 1).

#### I. Integrating theory into practice

The students perceived the debriefing session to be vital for reflection, enhancing their critical thinking and reasoning through discussions and feedback conducted with facilitators and fellow students. They perceived that facilitators’ questions generated fruitful and evolving discussions, highlighting correct and incorrect actions in SBE scenarios, thus stimulating reflection. This critical thinking and reflection were seen as beneficial for improving clinical knowledge and skills, and one student said:

[…] debriefing helps to know how everyone participated in the scenario, what he or she did well,you get to know your weakness. And you are asked why you did this which helps to recall back. So, I had more chances of developing knowledge, confidence, and skills of what I didn't do to the previous procedure (FGD-1, R1).

Students felt that facilitators enhanced their critical thinking and reasoning during debriefing, improving their knowledge and skills. This helped them to better judge their decisions and actions in neonatal care, especially when nurses were busy. One informant said:

Simulation debrief has improved our knowledge and practice through reflection and reasoning for why and whatever care we provided. Before simulation, we learned why we should dry and keep the baby warm, and wipe secretion from the baby’s mouth. In the busy clinical settings there is no one to teach us, because the nurses are busy and therefore they cannot explain to us what they are doing. So, through simulation, we can improve our practice with reasoning ability (FGD-1, R1).

Related to students’ reasoning ability rose in debriefing, they expressed to improve their ability to judge changes in neonates’ health and understood the importance of preventive measures for neonatal safety. This reasoning enhanced adherence to clinical standards. Students found debriefing vital to integrate and apply their theoretical knowledge to clinical practice. One informant said:

[.... ] In simulation I felt improved in confidence and reasoning as we were discussing how to do things and why we had to do that. So, after knowing why I should do this, I was able to maintain safety. Like we were asked why should you cover the baby? We had to reason…. to prevent hypothermia. So, we have improved confidence, reasoning, and adherence to clinical standards (FGD-1, R2).

#### II. Communication in neonatal emergency management

As a part of how the students experienced strengthened abilities for reasoning through simulation and the following debriefing, students also referred to how they were able to learn that clear and concise communication was vital for neonatal emergency management. In addition, they perceived that debriefing facilitated interprofessional teamwork cooperation during neonatal resuscitation through calling for assistance. This observation is well described by the following narrative:

Simulation has helped me to communicate well during practicing in the clinical area. I have got enough knowledge on how to distribute duties once the scenario happens in clinical area. Especially when the baby is not crying, so we called for help and shouted for help for assistance (FGI-2, R-11).

In summary, the students perceived that SBE strengthened their confidence crucial for clinical practice and professional life and reduced their fear of making mistakes in safe teaching environments. Moreover, debriefing enhanced students’ critical thinking and reasoning which is vital for quality care. The debriefing session moreover improved students’ communication skills which is vital for neonatal emergency management.

## Discussion

The students in this study perceived simulation as an important method for learning and to ensure quality care. The themes that emerged from the analysis were as follows: (1) strengthened confidence through practice in safe teaching environment and (2) enhanced critical thinking and reasoning in debriefing.

In general, strengthened confidence among nursing students through SBE is not a new finding as it is widely reported in the literature [26, 43, 44]. Previous research in Tanzania also found that SBE strengthened nursing students’ confidence and competence through practice in safe environment [10]. However, these findings are new and relevant particularly in the Tanzanian context where pre-service education often lacks opportunities for hands-on practice suggesting that SBE has the potential to enhance students' confidence in performing initial neonatal care [26, 41, 42]. The results of the current study are in line with a Lancet report that highlights the benefit of implementing “information technology innovations such as SBE to transform health professional education and health care systems”[16], suggesting that SBE might be a new strategy to reform methods of teaching in nursing education to enhance competences and quality health care system. Furthermore, Kruk et al.’s [43] report highlights that it is critical to provide knowledge and competence to students while in education. They particularly emphasize the importance of achieving competence through “active learning, early clinical exposure, and problem-based learning” [16], indicating that SBE during education, like carried out in this study, is of utmost importance to strengthen knowledge and competence among health care students. Thus, implementing SBE to enhance students’ procedural confidence is vital for preparing them to become skilled HCPs to improve the quality of neonatal care in contexts with high rate of neonatal mortality [17, 44].

The discussion will further focus on how students perceived SBE to enhance their critical thinking and reasoning during debriefing, and how SBE hence may bridge the theory–practice gap. Traditionally, many higher health education institutions in low-income settings underutilize pedagogic methods such as discussions, reflections, critical thinking, or reasoning, which are commonly used in SBE debriefing phase [10, 17]. Large auditorium lectures are widespread pedagogic method in these contexts—a method that provides minimal student activity [45].

The results of this study emphasize the importance of debriefing in fostering critical thinking and reasoning. During debriefing, facilitators lead the discussion and guided students through reflection, and feedback, allowing students to analyze both correct and incorrect performances from simulated scenarios. These discussions stimulate students’ reflections, critical thinking, and reasoning, thus, integrating theoretical knowledge with skills practice—crucial for patient safety [27, 41].

These observations align with existing literature emphasizing the importance of debriefing in SBE. Dreifuerst [46] highlights the impact of discussions and reflection in debriefing with an intention to enhance thinking and learning outcomes such as knowledge and skills. The students in the current study pointed out that debriefing helped them learn to integrate theory with practice through reflection and discussion based on the different simulated scenarios. This observation highlights the facilitators’ role in conducting debriefing following the six steps of Gibb’s reflective cycle [29], to support the discussion process by guiding students to analyze their experiences [29]. All six steps that guide students’ reflection process and learning outcomes are also stated by Husebø et al. [47].

The students reported that debriefing discussions helped them bring scenario experiences into focus. This was found based on their experiences of reflecting on what happened in the scenario and expressing how they learned from it. They communicated. They understand the rationale behind the rationale behind the clinical actions they carried out in the simulated scenario. This can be found to have improved their reasoning abilities and enhanced their adherence to clinical standard skills vital for ensuring patient safety and delivering high-quality neonatal care. Similar results by Guerrero et al. [27] and WHO [48] reported that students can reflect, contemplate, and relate their skills performance during the simulation to full interpretation that helps them to identify and create plans for application in the clinical settings.

The students in this study found debriefing to be an important setting for learning. According to the students, they were eager to receive corrections during debriefing for actions that were not properly performed in scenarios. The students argued that when they performed incorrect procedures and received feedback, they reinforced improvements and corrected their mistakes. These experiences align with several studies about how students experience the debriefing phase as a setting for clear feedback and reinforcement, rather than an evaluation of their performance [10, 49]. According to Dreifuerst and Drecker (2012), it is common for students to reflect on what went well as well as what went wrong during the debriefing phase to be able to act differently.

the next time. The students’ expectation of receiving corrections during the debriefing in this study might be linked to their positive view of SBE that train them in a safe environment.

In SBE, no real or alive patient gets hurt. The students in this study were simulating scenarios concerning initial neonatal care in a safe environment—unlike the hospital settings where the consequences of making mistakes in practice are detrimental. Skill practices in the simulation and feedback seemed to give the students confidence and assurance to guide and prepare them for safer clinical practice. Gegenfurtner et al. [50] state that the students’ intrinsic motivation employed to learn and apply knowledge are vital in the process of transfering theoretical knowledge into practice.

In the current study, the students were eager to practice initial neonatal care but faced challenges with the resuscitation equipment such as Ambu-bag and Apgar score chart to score the neonates’ skin color, heart rate, reflex irritability, muscle tone, and breathing, both checked at birth. This experience highlighted the importance of simulation training for knowledge transfer into practice. The purpose of knowledge transfer into practice is underlined in motivation theory and in Gibb’s reflective cycle (the conclusion and action plan steps 5 and 6), both aiming to prepare students for clinical practice and future skilled professionals [47, 50]. The results of this study stress the importance of SBE among students to enhance the transfer of clinical learning and quality care.

According to students in this study, SBE helped them to train skills and transfer knowledge in neonatal resuscitation practice. These results are in line with studies that reported SBE to improve confidence, skills, and proficiency in practice [51, 52]. Reflection on how students perceive SBE to enhance their confidence, critical thinking, and reasoning in a low-income context is highly important. The results from this study support that SBE is a pedagogical method for students’ learning which may transfer theory into practice [49, 53, 22]. The debriefing sessions also facilitated improved communication and teamwork among students, particularly in managing neonatal emergencies.

Students in this study reported that debriefing sessions improved their communication and teamwork for prompt actions in emergency neonatal situations. Effective communication was facilitated through calls, shouts for assistance, interactions, and role allocation during neonatal emergencies such as difficulty breathing at birth—skills critical for effective interprofessional collaboration. These actions in the scenarios align with previous studies that reported how SBE improves students’ communication skills and team collaboration [11, 54]. Tjoflåt et al. [11] and Hustad et al. [54] confirmed the students’ ability to detect a deteriorating patient, communicate within the team, and prioritize care, is vital in the management of emergency situations. The results from the current study emphasize the importance of preparing students for clinical practice through the implementation of SBE. Evidence of students' communication abilities can be found in simulation learning associated with debriefing sessions [47, 55].

### Limitation

This study was conducted in one urban diploma nursing and midwifery school in a low-income context, Tanzania. Therefore, transferability of the results of this study to other diploma nursing and midwifery schools in this context may be limited. SBE could be implemented to transform nursing education in countries with similar healthcare challenges. The study is potentially at risk of observer bias as the researcher was involved in teaching the participants. The participants' responses were tape-recorded and anonymized, ensuring they were not linked to individual identities, thereby reducing the likelihood of bias from the research team.

## Conclusion

Nursing and midwifery students perceived SBE as an effective method to prepare for clinical practice and quality neonatal care. Introducing SBE in nursing education may benefit students’ learning and strengthen the sustainability of skilled HCPs in low-income contexts where resources are scarce. Further research is needed to assess whether students can transfer knowledge into clinical skills practice.

## Data Availability

Data is available from the corresponding author on request.

## Abbreviations

HCPs: Health care providers
INC: Initial neonatal care
IRC: Institutional Review Committee
NatHREC: National Health Research Ethics Committee
SBE: Simulation-based education

## References

[1] Chabrera C, Dobrowolska B, Jackson C, Kane R, Kasimovskaya N, Kennedy S, et al. Simulation in nursing education programs: findings from an international exploratory study. Clin Simul Nurs. 2021;59:23–31.

[2] Sundler AJ, Pettersson A, Berglund M. Undergraduate nursing students’ experiences when examining nursing skills in clinical simulation laboratories with high-fidelity patient simulators: a phenomenological research study. Nurse Educ Today [Internet]. 2015;35(12):1257–61. Available from: 10.1016/j.nedt.2015.04.008 10.1016/j.nedt.2015.04.008 25943280

[3] Saeidi R, Gholami M. Comparison of effect of simulation-based neonatal resuscitation education and traditional education on knowledge of nursing students. Iran J Neonatol. 2017;8(2).

[4] Azizi M, Ramezani G, Karimi E, Hayat AA, Faghihi SA, Keshavarzi MH. A comparison of the effects of teaching through simulation and the traditional method on nursing students’ self-efficacy skills and clinical performance: a quasi-experimental study. BMC Nurs. 2022;21(1):1–8. 36261828 10.1186/s12912-022-01065-z PMC9581552

[5] Theobald KA, Tutticci N, Ramsbotham J, Johnston S. Effectiveness of using simulation in the development of clinical reasoning in undergraduate nursing students: a systematic review. Nurse Educ Pract [Internet]. 2021;57(October):103220. Available from: 10.1016/j.nepr.2021.103220 10.1016/j.nepr.2021.103220 34781195

[6] Sterner A, Sköld R, Andersson H. Effects of blended simulation on nursing students’ critical thinking skills: a quantitative study. SAGE Open Nurs. 2023;9. 10.1177/23779608231177566 PMC10201174 37223219

[7] Tansley G, Bailey JG, Gu Y, Murray M, Livingston P, Georges N, et al. Efficacy of surgical simulation training in a low-income country. World J Surg. 2016;40(11):2643–9. 27250083 10.1007/s00268-016-3573-3

[8] Livingston P, Bailey J, Ntakiyiruta G, Mukwesi C, Whynot S, Brindley P. Development of a simulation and skills centre in East Africa: a Rwandan-Canadian partnership. Pan Afr Med J. 2014;17:2–4. 10.11604/pamj.2014.17.315.4211 PMC4198314 25328611

[9] Jyoti, Devi AKM, Khushbu, Shalu. Simulation versus traditional method of teaching on the retention of birthing care. Indian J Forensic Med Toxicol [Internet]. 2021;15(3):276–83. Available from: http://medicopublication.com/index.php/ijfmt/article/download/15319/13730%0Ahttp://ovidsp.ovid.com/ovidweb.cgi?T=JS&PAGE=reference&D=emed22&NEWS=N&AN=2007792077

[10] Bø B, Madangi BP, Ralaitafika H, Ersdal HL, Tjoflåt I. Nursing students’ experiences with simulation-based education as a pedagogic method in low-resource settings: a mixed-method study. J Clin Nurs. 2022;31(9–10):1362–76. 34423486 10.1111/jocn.15996

[11] Tjoflåt I, Våga BB, Søreide E. Implementing simulation in a nursing education programme: a case report from Tanzania. Adv Simul. 2017;2(1):4–7. 10.1186/s41077-017-0048-z PMC5806360 29450018

[12] Mduma E, Ersdal H, Svensen E, Kidanto H, Auestad B, Perlman J. Frequent brief on-site simulation training and reduction in 24-h neonatal mortality-an educational intervention study. Resuscitation [Internet]. 2015;93:1–7. Available from: 10.1016/j.resuscitation.2015.04.019 10.1016/j.resuscitation.2015.04.019 25957942

[13] Ersdal HL, Vossius C, Bayo E, Mduma E, Perlman J, Lippert A, et al. A one-day “Helping Babies Breathe” course improves simulated performance but not clinical management of neonates. Resuscitation [Internet]. 2013;84(10):1422–7. Available from: 10.1016/j.resuscitation.2013.04.005 10.1016/j.resuscitation.2013.04.005 23612024

[14] Matterson HH, Szyld D, Green BR, Howell HB, Pusic MV, Mally PV, et al. Neonatal resuscitation experience curves: simulation based mastery learning booster sessions and skill decay patterns among pediatric residents. J Perinat Med. 2018;46(8):934–41. 29451862 10.1515/jpm-2017-0330

[15] Vadla MS, Moshiro R, Mdoe P, Eilevstjønn J, Kvaløy JT, Hhoki BH, et al. Newborn resuscitation simulation training and changes in clinical performance and perinatal outcomes: a clinical observational study of 10,481 births. Adv Simul [Internet]. 2022;7(1):1–11. Available from: 10.1186/s41077-022-00234-z 10.1186/s41077-022-00234-z PMC9636744 36335400

[16] Frenk J, Chen LC, Chandran L, Groff EOH, King R, Meleis A, et al. Health policy challenges and opportunities for educating health professionals after the COVID-19 pandemic. Lancet [Internet]. 2022;400(10362):1539–56. Available from: 10.1016/S0140-6736(22)02092-X 10.1016/S0140-6736(22)02092-X PMC9612849 36522209

[17] Tjoflåt I, Koyo SL, Bø B. Simulation-based education as a pedagogic method in nurse education programmes in sub-Saharan Africa – perspectives from nurse teachers. Nurse Educ Pract [Internet]. 2021;52(March):103037. Available from: 10.1016/j.nepr.2021.103037 10.1016/j.nepr.2021.103037 33839595

[18] Joho AA, Kibusi SM, Mwampagatwa I. Predictors of Helping Babies Breathe knowledge and skills among nurses in primary health settings in Dodoma region. Tanzania BMC Pregnancy Childbirth. 2020;20(1):1–7. 10.1186/s12884-020-2782-9 PMC7068969 32164561

[19] Meaney PA, Hokororo A, Ndosi H, Dahlen A, Jacob T, Mwanga JR, et al. Implementing adaptive e-learning for newborn care in Tanzania: an observational study of provider engagement and knowledge gains. BMJ Open. 2024;14(2):1–14. 10.1136/bmjopen-2023-077834 PMC10840034 38309746

[20] Shikuku DN, Milimo B, Ayebare E, Gisore P, Nalwadda G. Quality of care during neonatal resuscitation in Kakamega county general hospital, Kenya: a direct observation study. Biomed Res Int. 2017;2017. 10.1155/2017/2152487 PMC5682044 29214159

[21] WHO 2020. Simulation in nursing and midwifery education Simulation in nursing and midwifery education. 00(00):1–10.

[22] Kruk ME, Gage AD, Arsenault C, Jordan K, Leslie HH, Roder-DeWan S, et al. High-quality health systems in the Sustainable Development Goals era: time for a revolution. Lancet Glob Heal. 2018;6(11):e1196–252. 10.1016/S2214-109X(18)30386-3 PMC7734391 30196093

[23] Appiah-Kusi E, Christianson TM. Pediatric simulation in undergraduate nursing education: a scoping review. Int J Africa Nurs Sci. 2024;2023:10064. 10.1016/j.ijans.2023.100647.

[24] Alharbi A, Nurfianti A, Mullen RF, McClure JD, Miller WH. The effectiveness of simulation-based learning (SBL) on students’ knowledge and skills in nursing programs: a systematic review. BMC Med Educ [Internet]. 2024;24(1):1099. Available from: 10.1186/s12909-024-06080-z 10.1186/s12909-024-06080-z PMC11459713 39375684

[25] Chae S, Shon S. Effectiveness of simulation-based interprofessional education on teamwork and communication skills in neonatal resuscitation. BMC Med Educ. 2024;24(1):1–12. 38822320 10.1186/s12909-024-05581-1 PMC11143663

[26] Jeffries PR, Rodgers B, Adamson K. NLN Jeffries Simulation Theory : brief narrative description. 2015;292–3. 10.5480/1536-5026-36.5.292 26521496

[27] Guerrero JG, Tungpalan-Castro GM, Pingue-Raguini M. Impact of simulation debriefing structure on knowledge and skill acquisition for postgraduate critical care nursing students: three-phase vs. multiphase. BMC Nurs [Internet]. 2022;21(1):1–9. Available from: 10.1186/s12912-022-01100-z 10.1186/s12912-022-01100-z PMC9682710 36419144

[28] Gibbs G. Gibbs Reflective cycle. Jasper M [Internet]. 2020;(June):2–3. Available from: https://www.ed.ac.uk/

[29] Gibbs G. Gibbs’ (1988) reflective cycle. 1988;1–4.

[30] Coutinho VRD, Martins JCA, Pereira F. Structured debriefing in nursing simulation: students’ perceptions. J Nurs Educ Pract. 2016;6(9):127–34.

[31] Sahin G, Basak T. Debriefing methods in simulation-based education. J Educ Res Nurs. 2021;18(3):341–6.

[32] Egenberg S, Masenga G, Bru LE, Eggebø TM, Mushi C, Massay D, et al. Impact of multi-professional, scenario-based training on postpartum hemorrhage in Tanzania: a quasi-experimental, pre- vs. post-intervention study. BMC Pregnancy Childbirth. 2017;17(1):1–11. 10.1186/s12884-017-1478-2 PMC5584507 28874123

[33] Nelissen E, Ersdal H, Mduma E, Evjen-Olsen B, Broerse J, van Roosmalen J, et al. Helping mothers survive bleeding after birth: retention of knowledge, skills, and confidence nine months after obstetric simulation-based training. BMC Pregnancy Childbirth. 2015;15(1):1–7. 26303614 10.1186/s12884-015-0612-2 PMC4548347

[34] Campbell S, Greenwood M, Prior S, Shearer T, Walkem K, Young S, et al. Purposive sampling: complex or simple? Research case examples J Res Nurs. 2020;25(8):652–61. 34394687 10.1177/1744987120927206 PMC7932468

[35] McDermott DS, Ludlow J, Horsley E, Meakim C. Healthcare simulation standards of best PracticeTM prebriefing: preparation and briefing. Clin Simul Nurs [Internet]. 2021;58:9–13. Available from: 10.1016/j.ecns.2021.08.008

[36] Kjellsson G, Clarke P, Gerdtham UG. Forgetting to remember or remembering to forget: a study of the recall period length in health care survey questions. J Health Econ [Internet]. 2014;35(1):34–46. Available from: 10.1016/j.jhealeco.2014.01.007 10.1016/j.jhealeco.2014.01.007 24595066

[37] Graneheim UH, Lundman B. Qualitative content analysis in nursing research: concepts, procedures and measures to achieve trustworthiness. Nurse Educ Today. 2004;24(2):105–12. 14769454 10.1016/j.nedt.2003.10.001

[38] Riazi AM, Rezvani R, Ghanbar H. Trustworthiness in L2 writing research: A review and analysis of qualitative articles in the Journal of Second Language Writing. Res Methods Appl Linguist. 2023;2(3):1–14.

[39] Elo S, Kääriäinen M, Kanste O, Pölkki T, Utriainen K, Kyngäs H. Qualitative content analysis SAGE Open. 2014;4(1):215824401452263.

[40] Benites LC, Do Nascimento JV, Milistetd M, Farias GO. Content analysis in educational research in physical education: a study on curricular internship. Movimento. 2016;22(1):35–50.

[41] Al Gharibi KA, JA. Repeated Simulation Experience on Self-Confidence, Critical Thinking, and Competence of Nurses and Nursing Students—An Integrative Review. SAGE Open Nurs. 2020;6:1–8. 10.1177/2377960820927377 PMC7774432 33415282

[42] Bailey L, Emory J. High-fidelity simulation improves confidence in nursing students. Teach Learn Nurs. 2022;17(2):191–4.

[43] Kruk ME, Gage AD, Arsenault C, Jordan K, Leslie HH, Roder-dewan S, et al. The Lancet Global Health Commission High-quality health systems in the Sustainable Development Goals era : time for a revolution. 2018;6(November):1196–252. 10.1016/S2214-109X(18)30386-3 PMC7734391 30196093

[44] Kamath-Rayne BD, Thukral A, Visick MK, Schoen E, Amick E, Deorari A, et al. Helping Babies Breathe, second edition: a model for strengthening educational programs to increase global newborn survival. Glob Heal Sci Pract. 2018;6(3):538–51. 10.9745/GHSP-D-18-00147 PMC6172134 30287531

[45] Mwalabu G, Msosa A, Tjoflåt I, Urstad KH, Bø B, Furskog Risa C, et al. Simulation-based education to facilitate clinical readiness in nursing and midwifery programmes in sub-Saharan Africa: a meta-synthesis. High Educ Ski Work Learn. 2024;14(3):723–42.

[46] Dreifuerst KT. Getting started with debriefing for meaningful learning. Clin Simul Nurs [Internet]. 2015;11(5):268–75. Available from: 10.1016/j.ecns.2015.01.005

[47] Husebø SE, O’Regan S, Nestel D. Reflective practice and its role in simulation. Clin Simul Nurs [Internet]. 2015;11(8):368–75. Available from: 10.1016/j.ecns.2015.04.005

[48] WHO. Simulation in nursing and midwifery education Simulation in nursing and midwifery education. Copenhagen: WHO; 2018.

[49] Botma Y. Nursing student’s perceptions on how immersive simulation promotes theory-practice integration. Int J Africa Nurs Sci. 2014;1(December):1–5.

[50] Gegenfurtner A. Dimensions of motivation to transfer: a longitudinal analysis of their influence on retention, transfer, and attitude change. Vocat Learn. 2013;6(2):187–205.

[51] Alalhareth N, Howarth M. The Effectiveness of Simulation Training on Nursing Students’ Neonatal Resuscitation Skills: A Systematic Review. Int J Nurs Heal Care Res. 2020;03(07):1–17.

[52] Mishra R, Hemlata, Trivedi D. Simulation-based learning in nursing curriculum- time to prepare quality nurses: A systematic review and meta-analysis. Heliyon. 2023;9(5):e16014. Available from: 10.1016/j.heliyon.2023.e16014. 10.1016/j.heliyon.2023.e16014 PMC10189473 37206022

[53] Wong SHV, Kowitlawakul Y, Exploring perceptions and barriers in developing critical thinking and clinical reasoning of nursing students: a qualitative study. Nurse Educ Today. 2020(95) 104600. Available from: December 2019. 10.1016/j.nedt.2020.104600. 10.1016/j.nedt.2020.104600 32992269

[54] Hustad J, Johannesen B, Fossum M, Hovland OJ. Nursing students’ transfer of learning outcomes from simulation-based training to clinical practice: a focus-group study. BMC Nurs. 2019;18(1):1–8. 31719793 10.1186/s12912-019-0376-5 PMC6839188

[55] Reigeluth CM, editor. Instructional- Design Theories and Models Volume II A New Paradigm of Instructional Theory. Vol 2. New York: Lawrence Erlbaum Associates; 1999. p. 1–74.

